# Public Preferences in Resource Allocation for Insurance Coverage of Dental Implant Service in South Korea: Citizens’ Jury

**DOI:** 10.3390/ijerph18084135

**Published:** 2021-04-14

**Authors:** Hwa-Young Lee, Eun-Young Bae, Kyungdo Lee, Minah Kang, Juhwan Oh

**Affiliations:** 1 Department of Global Health and Population, Harvard T.H. Chan School of Public Health, Boston, MA 02115, USA; diana0224@gmail.com; 2 Institute of Convergence Science (ICONS), Convergence Science Academy, Yonsei University, Seoul 03722, Korea; 3 College of Pharmacy, Gyeongsang National University, Jinju-si 52828, Korea; eybae@gnu.ac.kr; 4 Department of Health Behavior, Society and Policy, Rutgers School of Public Health, Piscataway, NJ 08854, USA; kd.lee.md@rutgers.edu; 5 Department of Public Administration, Ewha Womans University, Seodaemun-gu, Seoul 120-750, Korea; minahkang@ewha.ac.kr; 6 Department of Medicine, Seoul National University College of Medicine, Seoul 03080, Korea

**Keywords:** priority setting, Citizen’s Jury, dental implant service

## Abstract

The Korean government sought to include dental implant services for the elderly in the benefits package of the national health insurance. In 2014, the Citizens’ Jury was held to discuss the topic, during which thirty jurors, randomly selected from the 2665 applicants, participated in a day-long deliberation process after having an information session on the topic by a team of experts. There was a substantial shift in opinion during the deliberation session toward a more cost-conscious view. Most jurors supported limiting the coverage of dental implant to only one tooth per individual given the extent of the financial burden that will be imposed on the population. They opposed covering implant services for the front teeth, given that the implant of front teeth generally serves aesthetic purposes rather than restoring mastication function. The government’s final decision in 2014 was to offer coverage up to two teeth, regardless of tooth location. This scheme based on the jury’s recommendations in 2014 has been implemented without policy failure to date, which shows that the lay public can meaningfully contribute to a decision-making process regarding controversial agendas such as benefits packages for expensive health services.

## 1. Introduction

The increasing age of the population and growing expectations of people for a healthy life have escalated the demand for dental care services. Tooth loss among the elderly is one of the biggest contributors to impaired oral health, which affects physical health through the reduced capacity for chewing. It has also been shown to deteriorate cognitive function [1,2]. Previous studies have demonstrated a positive association between tooth loss and increased risk of dementia, depression, and anxiety in adults [3,4]. Although there has been a mounting demand for insurance coverage of services for tooth replacement, insurance benefit in dental care services in South Korea has been limited to the most basic services such as treatments for cavities and gum disease, and tooth extraction for numerous decades. Only recently, the coverage was expanded to cover cavity filling for children less than 12 years old (from the year 2009) and dentures for the elderly (from 2012). This in turn has resulted in an excessive financial burden for service-users. According to the data, the average cost for implant service (1066~1416 thousand KRW (≈955~1255 USD)) amounted almost to the average monthly income of the elderly (equivalized disposable income for the elderly ≥65 was 1140 thousand KRW in 2013 (≈1101 USD)) [5,6].

This circumstance has led one of the leading candidates (and ultimately elected) in the election for the 18th President of Korea to publicly promise insurance coverage expansion for dental implant service for the elderly over 65 years of age. However, this was criticized as a political move rooted in populism to appeal to the elderly voters and therefore faced strong opposition. However, the President continued to pursue the promise after the inauguration in 2013 with a plan to start coverage of dental implant services from 2014 for the elderly aged more than 75 with 50% co-payment and to extend by lowering eligible age for benefit gradually from 75 to 70 in 2015, and 65 in 2016.

### 1.1. Health Insurance Coverage of Dental Care Services

Although Korean health insurance achieved universal population coverage from its early stage, its coverage scope has remained narrow [7]. Thus, the government initiated to expand health insurance coverage as a national agenda in 2005 and continued its effort ever since.

The overall decision-making mechanism for benefit expansion can be summarized as follows; A new medical technology is applied to be included in the health insurance benefit package by the manufacturer or service provider. The proposal is then reviewed by the Health Insurance Review and Assessment Service. Separately, candidates to be included in the benefit package among the pre-existing uninsured services or items, i.e., those currently being paid out-of-pocket, are selected and reviewed by National Health Insurance Service (NHIS) [8]. The final decision for coverage is decided by the Health Insurance Policy Deliberation Committee (HIPDC) within the Ministry of Health and Welfare (MoHW), which is composed of payers, providers, public agencies, and technical experts [6]. Although there has been an effort to secure fair decision-making on what should be prioritized in insurance coverage expansion, e.g., developing guidelines for prioritization and making a list of the service items to be covered prioritized by the guidelines, it is unclear how such guidelines or list have been utilized. Not an inconsequential number of decisions have been made politically without a thoughtful priority-setting process, and coverage of dental implant service was one of such decisions.

Most countries are not generous in providing coverage of dental prosthetic services by public financing scheme even when they are for therapeutic purposes. This is partly because they are costly and therefore have a considerable impact on the insurance budget although they neither treat nor mitigate life-threatening conditions. Denture service is covered in some countries such as the US, Canada, Australia, Sweden, etc., mostly for the targeted sub-population and with shallow coverage [9]. Coverage for implant services is even rarer. In Sweden, one of the most generous countries regarding its health and welfare policies, payments for the dental implant are subsidized through a special high-cost protection scheme since 2012 [10]. The UK also provides coverage through a special subsidy, which depends on the clinical commissioning group [6,11,12].

Although dental implant was considered cost-effective when compared to the fixed partial denture in South Korea [13]. due to its potential impact on the insurance budget and the lack of precedent for public funding of implant services for the population in other countries NHIS can refer to, there was tension around the details of coverage decision for dental implant among government, public, and professional societies [14]. Finally, NHIS, a single-payer of Korean National Health Insurance, decided to hold a Citizens’ Jury in 2013 to elucidate our collective social value on the issue.

### 1.2. Citizens’ Jury in Health Insurance Priority Setting in South Korea

Priority setting in health involves not only “technical” judgment such as clinical efficacy or cost-effectiveness captured by scientific evidence but also incorporates “social value” judgment [15]. Social value judgment may vary depending on moral or ethical values that members of a particular society uphold [16,17]. The social value would vary across different societies due to different backgrounds in politics, culture, social demographics, religions, and economic status, rendering social value distinct from a purely moral value that is commonly held across different societies [16]. Social values are not easily delineated by mere technical criteria. Public participation in healthcare decision-making can therefore ensure that social value judgments regarding healthcare reflect the opinions of the “lay” public [18].

Korean NHIS held the first experimental Citizens’ Jury in 2008 as a mechanism to involve the lay public in decision-making in health insurance coverage with the hopes to improve the health insurance governance [18]. The Citizens’ Jury is basically a deliberative process where sufficiently informed citizens pursue a careful and serious weighing of reasons for and against certain propositions [19]. NIHS organized a meeting, aiming to adhere to a conceptual framework for priority-setting of “accountability for reasonableness” proposed by Norman Daniels [20]. After three rounds of experimental meetings in 2008, 2010, and 2012, it has been held every year as an official mechanism for public participation to discuss a range of topics regarding expanding the benefit coverage. Specific agendas put to each meeting have been selected by NHIS among the ones requested by MoHW.

Although several countries (i.e., Canada, U.K, or Australia) have used public deliberation to seek out social values in diverse topics in health care priority setting, public preference for priority setting regarding implant service for the elderly has never been discussed in any countries to date. This study aims to examine the process, the findings, recommendations of the Citizens’ Jury on the optimized resource allocation for dental implant service to provide an example for other countries in consideration of the public coverage of dental implant services.

## 2. Method

### 2.1. Recruitment and Selection

Participants were recruited by public advertisement through the NHIS website, media specialized in healthcare, local newspapers, and major Social Network Services for 15 days (11–20 February 2014). The application was open to all Korean citizens older than 19 years old. Interested applicants applied through the website, email, fax, postal service, and phone. The applicants were asked to provide information on basic demographic and socioeconomic status such as gender, age, job, residential area, educational attainment, disability status, and history of chronic disease. A total of 2665 applied, 93% of which applied through website, 3.5% email, 1.9% fax, 1.5% phone, and 0.1% postal service. 784 applicants who were affiliated with special interest groups, such as patient advocacy groups and those employed in healthcare sectors (medical institutions, pharmaceutical companies, or medical equipment companies, etc.) were excluded. Among the remaining 1881, a total of 30 eligible applicants were selected by gender-, age-, and residential region-stratified random sampling to match the demographic profile of the Korean population (within ±3 difference between expected frequency and observed frequency). Two were additionally selected from a group older than 65 to ensure that the beneficiary group of insurance coverage of the implant service was well-represented (Table 1). There ultimately were twenty-nine participants in the Citizens’ Jury with three absentees. An honorarium of 100,000 Korean Won (≈85 US $) for all participants and additional travel fees were provided to those traveling from areas other than Seoul.

### 2.2. Citizens’ Jury Process

Three sub-questions under one overarching question developed by the NHIS were posed for the Citizen’s Jury (Box 1).

The Citizens’ Jury was convened on 8 March 2014 in the meeting room of the NHIS located in Seoul, South Korea. The jurors were informed of the purpose of the process, which was to make a recommendation on the acceptable coverage scope of implant service. They were also told that their recommendation would be documented and delivered to the HIPDC for the final decision.

In the morning session, jurors were provided with key information on the three topics from three expert witnesses: budget status and financing mechanism of NHI, basic knowledge of dental implant procedure, and overall information on insurance coverage of dental implant including budget impact. Jurors were encouraged to ask questions about the contents delivered. In the afternoon, jurors participated in two deliberative sessions which lasted 100 min, each with a 20-min-long intermission, during which jurors freely shared their opinion, value, and relevant experience with each other. One facilitator assisted jurors in their thorough and open discussion on the issue while keeping the deliberation process fair and inclusive.

The poll on the overarching question and further sub-questions were conducted three times: before the outset of the whole process (pre); between the end of the learning session in the morning and the beginning of the deliberation in the afternoon (mid); and at the end of the deliberation (final). Participants were also encouraged to describe freely why they chose those answers. A satisfaction survey about the Citizens’ Jury process was completed at the end of the Jury. The stenographer was hired and present during the entire process. The jurors’ poll results on each question as well as rationales for the opinions were summarized by NHIS on behalf of the jurors and submitted to the HIPDC in a document for the final decision.

Box 1 **Questions put to the jury.**

Is there a need to limit the coverage scope of the dental implant service?

(1) The number of implants to be covered per individual.
  (1-1) Do you agree not to limit the number of covered implants?
  (1-2) Do you agree to limit the number of the covered implants to 1, 2, or 3?
(2) For which part of the teeth, the dental implant to be covered (front teeth vs. molar).
(3) Whether to cover implant for those who have already received the coverage benefit of a partial denture.

## 3. Results

The composition of the Citizens’ Jury reflected that of the Korean population. The intentional addition of two elderly to the original composition made the male, the unemployed, the higher educated, and the non-disabled slightly over-represented compared to the Korean population (Table 1).

### 3.1. Overarching Question: Is There a Need to Limit the Coverage Scope of the Dental Implant Service?

In pre-poll, 75.0% of jurors agreed to place a limit on the coverage scope of dental implant services such as the age of beneficiary or the number of teeth to be covered. The share remained the same in the mid- and final-poll (75.0%).

#### 3.1.1. Sub-Question 1: How Many Implants to Be Covered per Individual?

**Figure 1:**
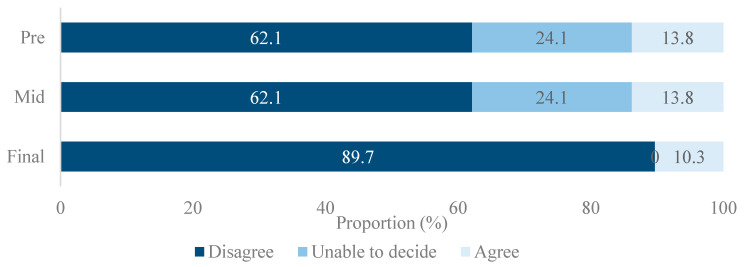
The proportion of jurors who disagreed not to limit the number of implants to be covered in pre-, mid-, and final survey.

The proportion of jurors that disagreed with unlimited implant coverage was 62.1% in both pre- and mid-poll surveys, which arose to 89.7% in the final poll after deliberation (Figure 1). The proportion of jurors who were unable to decide decreased from 24.1% in the pre- and mid-poll to 0% in the final poll (Figure 1). All jurors in the 19~39 age group and 87.5% among jurors older than 60 years disagreed with the no-limit option (Results not shown). Male jurors (93.8%) showed more disagreement than female (84.6%) (Results not shown).

**Figure 2:**
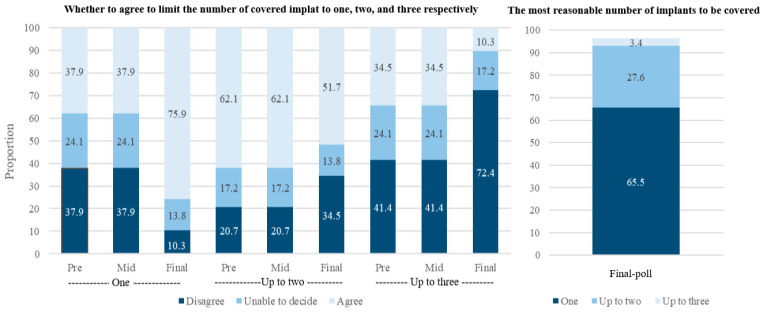
The poll results on the number of dental implants to be covered.

Jurors were asked to answer whether they agreed to limit the number of covered implants to one, two, or three respectively. In the pre- and mid-poll, the proportion of jurors that agreed to cover up to two was the highest (62.1%, the same in pre- and mid-poll). In the final poll, however, the proportion of jurors that agreed to cover only one implant turned out to be the highest (75.9%, 51.7%, and 10.3% for one, two, and three, respectively). When directly asked to choose only one option, the most reasonable number of coverage was one (65.5%), followed by two (27.6%). Only 3.4% answered that coverage up to three was acceptable (Figure 2).

The most frequent rationale presented during the deliberation was that unlimited coverage would result in too big of a burden on the NHI budget, especially considering the continuing increase in the elderly population. This would in turn lead to a continuous increase in premiums, ultimately resulting in a greater financial burden on the population. Jurors gave an opinion, to begin with, minimum coverage scope and to expand incrementally later, if needed, since delisting the service from the benefits package would be much more challenging than vice-versa. There were also other concerns that unlimited coverage may generate provider- or patient-induced demand whereas low-income groups still would not have access to dental implant services due to high co-payment (50%), which may worsen the inequality over time. Some jurors claimed that the implant service should not be covered at all because coverage of therapeutics for more life-threatening diseases are still suboptimal. They emphasized the importance of education or a campaign for preventive interventions for oral health rather than expansion of coverage to the dental implant service.

#### 3.1.2. Sub-Question 2: For Which Part of Teeth, the Dental Implant to Be Covered (Front Teeth vs. Molar)

In the pre-poll, 62.1% of participants answered that the implant should be covered regardless of the site while only 17.2% answered that coverage needs to be limited to the molar. On the other hand, in the final poll, 69.0% agreed to cover only the molar while only 27.6% agreed to cover both. The main reason for opposing cover for implants for the front teeth was that dental implants for front teeth are more or less for aesthetic purposes rather than function restoration while implant for molar teeth is mainly for restoring mastication function. They argued that health insurance coverage should be only for essential medical services. On the other hand, there was an opinion that limiting the number would be enough, and an additional limit to the site would overly restrict people’s choice. There was an additional concern that the said restriction is unfair for those who lost the front teeth only.

#### 3.1.3. Sub-Question 3: Should Implant for Those Who Already Have Benefited Insurance Coverage of Partial Denture Be Covered or Not?

**Figure 3:**
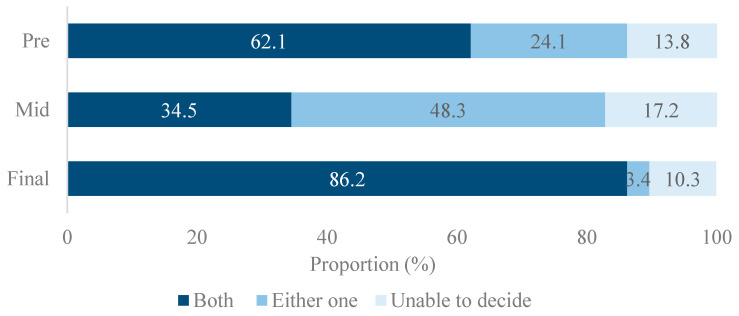
The proportion of agreement to double- coverage for partial denture and implant services on the same jaw in pre-, mid-, and final survey.

Response pattern to the question on additional coverage of implant for those who have already been provided insurance coverage of partial denture fluctuated throughout the sessions (Figure 3). The proportion of juror supporting duplicate coverage of the implant and partial denture on the same jaw was 62.1% in pre-poll. This decreased to 34.5% in the mid-poll and rose again to as high as 86.2% in the final poll. The proportion that agreed to dual coverage was the lowest among jurors older than 60 (75.0%) (Figure 3).

Rationales of jurors who agreed to allow the duplicated coverage of partial denture and implant were that medical need for both implant and partial denture on the same jaw (either upper or lower jaw) is a frequent clinical presentation. Disallowing duplicated coverage would also lead those who have already benefited from insurance coverage of partial denture to lose their option to choose between a partial denture and dental implant. In contrast, opponents argued that since repeated coverage of partial denture on the same jaw is currently not allowed, the same rule should be applied.

### 3.2. Jurors’ Feedback for the Participation Process

74% and 83% of jurors responded that the information provided by experts in the learning session was unbiased and adequate to understand the issue. Most (86.2%) of the jurors considered the duration of deliberation to be appropriate, and 75.9% responded that they were satisfied with the overall process. When asked about the capacity or potential of the lay public to contribute to the decision making of health insurance policy, more than half (58.6%) responded that the lay public can play a crucial role in policy decision making, followed by 37.9% of jurors responding that the public can play a role as reference or consultation group. Only 3.4% responded that they cannot play any role due to lack of expertise.

## 4. Discussion

To our knowledge, this case is the first to involve the public in decision-making in the coverage scope of dental implant service not only in South Korea but also worldwide. After deliberation, the Citizens’ Jury supported that the coverage of dental implant should be limited to one maxillary tooth only. In 2014, the HIPDC made a final decision that coverage of implant for the elderly aged more than 75 years would be limited up to only two teeth without site restriction with a plan to incrementally lower the age criteria of the target population to 70 in 2015 and 65 in 2016. Despite the initial concern, the actual expenditure of the insurance budget was less than estimated. It was forecasted that in 2015, about 3 trillion KRW (approximately 2.8 billion USD) would be spent for covering dental implant service for up to two teeth with allowing duplicated coverage with a partial denture. However, available data showed that actual expenditure for coverage of denture and implant combined was 2.7 trillion KRW in 2015 (approximately 2.5 billion USD) [21] and 2.2 trillion KRW in 2018 [22].

The Citizen’s Jury case in South Korea may have several important implications worthy of careful consideration for other countries. First, our case demonstrated that the lay public were able to consider the cost and benefit in a balanced way during deliberation. They, not only as a potential beneficiary but also an ultimate payer, were sensitive to both aspects. Jurors were largely in favor of limiting the benefit coverage of dental implants to only one tooth per individual on grounds of the lack of justification in spending such a big insurance budget for covering a relatively low-priority service. Usually, it is not users but payers who are more attentive to the cost. The public has been alleged to prefer to have benefits as much as possible with as little contribution as possible. However, the decision from our Citizens’ Jury may indicate that they can judge an agenda from a cost-benefit perspective through deliberation even without expertise on the complex concept of cost-effectiveness.

Second, the jury’s recommendation to limit the scope of coverage site only to molar teeth, whose main functional role being mastication, showed that the public prefers to spend the limited resource on services for improving health over services for cosmesis. This may indicate that they value collective duty to mutually support health conditions than satisfying personal preference.

Third, allowing a duplicated coverage of the implant and the partial denture may indicate that jurors are equity-sensitive. The rationale for their decision was that those who already had utilized a denture for missing teeth when insurance coverage for implant was not offered also should be allowed to choose an insured implant service if needs occur again. Although people with egalitarian minds usually tend to put less weight on the effectiveness or cost-effectiveness [23], it was found that our jury considered efficiency in conjunction with equity.

Fourth, a substantial change in the jury’s opinion during the deliberation session also warrants our attention. The proportion of jurors who opposed covering an unlimited number of implants substantially increased after the deliberation while it remained the same after the initial information session. In the information session, information on the impact on the health insurance budget to cover implant service was provided in an accessible and digestible way to laypeople. For example, it was explained how much more they need to pay an insurance premium when they allow coverage of one-, two-, and three implants per individual or what other service coverage they would have to forego if they do not want to raise insurance premium. Although this might have given the jury a chance to consider the trade-off or opportunity cost from their perspective, the jurors might have needed some time (deliberation) to digest this information since they may have hardly had a chance to allocate such a large amount of money in their daily lives. Additionally, during deliberation, participants could deeply think of which option is better for all others by exchanging preferences, opinions, reasonings, and values beyond (but based on) the information obtained during the information session [24]. It indicates that simply asking the public’s opinion without giving them chance to deliberate on the agenda may not reveal a true social value, especially in policy decisions involving significant budget consumption like a dental implant.

### For Deliberation to Be Fairer

Since 2014, NHIS has become more confident in the value of Citizens’ Jury, and therefore, has been holding it annually as a robust mechanism for public input in insurance policy decision making. Meanwhile, the Citizens’ Jury has been evolved with a few important changes. First, the Jury has been newly recruited every year at first, but the term was extended to two years in 2017. This improved the deliberation quality by enabling the juror to get used to the system and also raised the efficiency by saving the effort regarding time and money spent on the recruitment process. Second, they expanded the scope of topics, ranging from micro-topics such as “coverage of high-priced cancer drug” to more macro-topics such as “appropriate coverage level of the health insurance”. However, despite these efforts, there still exists much room for improvement to establish a fair process. First, while Abelson (2003) and Dukhanin (2018) presented how many jurors can have opportunities to input their opinions at the agenda-setting stage as one of the important principles when evaluating the fairness of the public participation process [19,25] the agenda in our jury was selected by the government. In addition, questions asked in our deliberation were too specific, requiring the jurors to think about more of a technical aspect rather than a value judgment. Our citizens’ jury was supposed to deliberate on details of coverage scope while whether or not the implant service would be covered was a fixed and unchangeable premise. The target population older than 75 years and a 50% co-payment rate were also predetermined by the government. Admittedly, if our jury was supposed to deliberate freely on these topics, they might not have agreed to the coverage of dental implants at all or might have made different decisions on the target population or co-payment rate.

Second, the citizens’ jury was one day long. Although the majority of jurors considered the duration of deliberation was enough, this was much shorter compared to the recommended 4–5 day-long time frame for public deliberation by Jefferson Center (2004) [26] or the UK institute for Public Policy Research [27]. This time constraint might have limited opportunity for full reflection on the preferences, values, and interests of others. Finally, we could not find any official record to confirm how the citizens’ jury’s input was incorporated into the final decision of HIPDC. The extent to which public input was incorporated into the final decision and how public input into the final decision was communicated to the public are considered important elements for a fair process [19].

## 5. Conclusions

The Citizens’ Jury’s decision offered momentum to the incremental expansion of implant service coverage in South Korea. The benefit scheme of dental implants based on the jury’s recommendation in 2014 has been implemented without policy failure or critical critique to date. This may have an important implication that the public can make an important contribution to reasonable decision-making through deliberation in a decision-making process on a controversial plan to cover a medically non-essential service (mostly un-insured in other similar economies). We expect that Citizen’s Jury for a dental implant in South Korea may serve as a good reference for other countries considering the government’s funding for dental prosthetic services. There, however, remains many gaps to be filled for a fairer deliberation process, especially in a mechanism of agenda-setting and communicating the jury’s decision with the public and the final decision-makers.

## Figures and Tables

**Table 1:** Description of Citizens’ Jury and comparison with the Korean population.

Criteria	Categories	Applicants (%)		Participants	
				Sample Frequency ^†^	Expected Frequency
Gender	Male	1684	(63.2)	15 (+2)	15
	Female	981	(36.8)	15	15
Age (years)	19~39	606	(22.7)	11	11
	40~59	1505	(56.5)	13	13
	≥69	554	(20.8)	6 (+2)	6
Residentialarea	Metropolitan area	1885	(70.7)	15 (+1)	15
	Other areas	780	(29.3)	15 (+1)	15
Job	Manager, professionals	694	(26)	4	4
	Clerical, service workers, sales, agriculture and fishing, technician, manual, military	1971	(74)	17	17
	Unemployed: students, housewives, retired, non-employed	898	(33.7)	9 (+2)	9
Educational attainment	Less than college graduate	1081	(40.6)	16	16
	College graduate and above	686	(25.7)	14 (+2)	14
Disability	Yes	761	(28.6)	2	2
	No	1138	(42.7)	28 (+2)	28
Chronic disease	Yes	766	(28.7)	5 (+1)	5
	No	612	(23)	25 (+1)	25

^†^: Figures in parenthesis are the number of additionally-added elderly participants.

## Data Availability

The data are available on a request basis.
